# Work and health: We need to focus on people not institutions

**DOI:** 10.1016/j.puhip.2024.100561

**Published:** 2024-12-10

**Authors:** Michelle Black, Paul Crawshaw, Amy Barnes

**Affiliations:** Department of Public Health Policy and Systems, University of Liverpool, Liverpool, UK; Public Policy, Teesside University, Middlesbrough, UK; Public Health and Society, Health Sciences, University of York, UK

Recent concerns that the parlous state of the people's health is a drag anchor on economic growth surface an age-old public health conundrum: *health selection* versus *social causation* as determinants of poor health and inequalities. Consensus has emerged that dwindling labour market participation is the cause of sluggish economic growth, with long-term sickness identified as the main determinant of historic and stubborn economic inactivity. As more people become and remain absent from labour markets, the modern conundrum becomes; does good health enable work or is good work vital for good health? Given the high rates of economic inactivity in working aged people across many high-income countries [1], this is an urgent question for policy and public health communities alike [2]. Governments everywhere need working age people to work, they are the engine of the economy. This is a specific challenge in the UK. With 9 million people economically inactive, the engine is asleep.

Economic inactivity is a status of being ‘*not in employment or seeking work within the last 4 weeks and unavailable to start work in the next 2 weeks’.* [3] For working age people economic inactivity in, for example, the UK has been steady to increasing over the last 30 years and now stands at 22 % [4,5]. Long term ill-health is one of the main reasons [5].

This is perhaps one reason why the newly elected UK government is investing over £20 billion into the NHS, recognising the ‘health selection’ argument long favoured by economists; long term ill-health is a barrier to work. Additionally, it has recently announced £240 million to support people with ill health and get Britain working [6](via skills and integrated health and employability support), a tentative recognition of ‘social causation’; work leads to good health.

Whilst not doubting that the health service needs resource, from a public health perspective the balance of investment here is perhaps misguided in terms of what it hopes to deliver. Given the evidence for social causation - that an estimated 47 % of our health is determined by social and economic conditions - and only 16 % by clinical care [7], the policy direction pursued in the UK, as in many other nations is clearly missing an opportunity. This is not a new phenomenon. Prevention may be better than cure, but the imperative to provide services that are there for us when we need them is a powerful one for governments.

Understanding the impact of good work on health and health inequalities should lead to policy decisions which actively promote a healthy labour market and favourable conditions for business growth. The recent UK White Paper, *Get Britain Working* [6], shows some promise, through explicit recognition of the complex two-way relationship between work and health and the potential benefits of increased labour market participation for both people and the economy.

If applying a true public health lens to this goal, the objective should be to focus on prevention by prioritising healthy and thriving *populations* who can access good work, above a narrow focus on whether the health service as an *institution* is fit for the future. It also involves asking whether the economy, as another key institution, is fit for purpose? Are current rules, norms and incentives within the economy set up to meet people's needs and deliver quality of life?

Action to improve health services and increase labour market participation is clearly a good thing, but a narrow focus on existing institutions can lead us down dystopian rabbit holes with people living with obesity seen as a ‘fiscal burden’ and justifying initiatives to give out weight loss drugs to ‘get unemployed people’ with obesity ‘back to work’ [8]: (*don't worry about unhealthy food environments or living conditions), eat what you like, the state will get you ‘healthy’ and working again, all is good!*

Current UK policy needs more ambition if it is to stir the engine of the economy. Although a focus on people and the social and economic conditions which shape life and opportunities to work are welcome, investment continues to be skewed heavily in favour of services delivered for treating the sick. Taking a public health lens to increasing economic activity demands a focus on both health selection, recognising it as a social issue [9] without discriminating against poor health, as well as social causation, the complex conditions structuring and entrenching inequalities. This means building a wellbeing economy centred on people [10], incentivising work and supporting employers to make environments accessible for those with complex and fluctuating long-term health conditions. It means recognising the impact of insecure work, the quality of work and the importance of inclusive access to work particularly for an ageing workforce [11,12]. Real ambition requires moving beyond the traditional orbit of public health, supporting colleagues in schools and colleges, training providers and workplaces to deliver a generational plan for skills development throughout people's lifecourse and education that prioritises a healthy population, willing and able to participate in good work. It means focusing on society and enabling people to thrive.
